# Uptake, translocation, and metabolization of amitriptyline, lidocaine, orphenadrine, and tramadol by cress and pea

**DOI:** 10.1007/s11356-024-32379-x

**Published:** 2024-02-16

**Authors:** Anna Detzlhofer, Christian Grechhamer, Lawrence Madikizela, Markus Himmelsbach, Franz Mlynek, Wolfgang Buchberger, Christian W. Klampfl

**Affiliations:** 1 Institute of Analytical and General Chemistry, Johannes Kepler University, Altenberger Strasse 69, 4040 Linz, Austria; 2 Institute for Nanotechnology and Water Sustainability, College of Science, Engineering and Technology, University of South Africa, Florida Science Campus, Roodepoort, 1710 South Africa

**Keywords:** Environmental analysis, Plant metabolism, Pharmaceuticals, Drift-tube ion-mobility mass spectrometry, Plant uptake

## Abstract

The uptake, translocation, and metabolization of four widely used drugs, amitriptyline, orphenadrine, lidocaine, and tramadol, were investigated in a laboratory study. Cress* (Lepidium sativum* L.) and pea (*Pisum sativum* L.) were employed as model plants. These plants were grown in tap water containing the selected pharmaceuticals at concentrations ranging from 0.010 to 10 mg L^−1^, whereby the latter concentration was employed for the (tentative) identification of drug-related metabolites formed within the plant. Thereby, mainly phase I metabolites were detected. Time-resolved uptake studies, with sampling after 1, 2, 4, 8, and 16 days, revealed that all four pharmaceuticals were taken up by the roots and further relocated to plant stem and leaves. Also in these studies, the corresponding phase I metabolites could be detected, and their translocation from root to stem (pea only) and finally leaves could be investigated.

**Supplementary Information:**

The online version contains supplementary material available at 10.1007/s11356-024-32379-x.

## Introduction

Increasing knowledge in medicine coupled with the ongoing development and release of new pharmaceuticals results in a continuous increase in the consumption of drugs for human and veterinary use. This trend is magnified by the fact that more and more people populate this world, and (luckily) an increasing percentage of the population gained access to medical treatment. Furthermore, the aging population (particularly in countries of the so-called “Western world”) even intensifies this process. Regrettably, this is affected with a negative side effect, namely the increasing release of pharmaceutically active substances (APIs) into the (primarily aquatic) environment (aus der Beck et al. 2016). One common route for contamination of surface waters with pharmaceuticals and personal care products (PPCPs) is their introduction via the municipal wastewater system. Despite the fact that water is treated in wastewater treatment plants (WWTP), analysis of their effluents revealed that still a range of substances is not fully removed from the wastewater and is subsequently released into rivers or lakes. In addition, sewage sludge from WWTP or manure from treated animals may be in use as fertilizers in agriculture thereby representing another source of contamination (Heberer 2002). Also, improper discharge from industrial sites manufacturing APIs needs to be considered in regions with a density of pharmaceutical industry (Wang et al. 2021). For this reason, within the last four decades, a large number of studies on the presence of PPCPs (and their metabolites) in environmental waters has been published (Adeleye et al. 2022; Anand et al. 2022; Bavumiragira et al. 2022; Gani et al. 2021); Hernández-Tenorio et al. 2022; Madikizela and Ncube 2021; Rathi et al. 2021; Rodrigues dos Santos et al. 2022).

In addition to the contamination of water, also its effect on plants and wildlife should be considered. It is a well-known fact, that plants, when brought into contact with water containing PPCPs, can take up these substances and translocate them from roots into stem, leaves, and fruit (if available) (Bartrons and Peñuelas 2017; Fu et al. 2019; Keerthanan et al. 2021; Madikizela et al. 2018; Pico et al. 2017; Reddy Pullagurala et al. 2018). After uptake, the parent drugs may subsequently be transformed into metabolites by the plant (Klampfl 2019; Klampfl et al. 2021; Sauvetre et al. 2021). Phase I metabolites, mostly formed by hydroxylation of the parent drug, may be further bio-transformed by conjugation with for example sugars, small organic acids, or amino acids (Emhofer et al. 2018, 2019; Fu et al. 2017; Kodešová et al. 2019; Riemenschneider et al. 2017). This issue is commonly studied by either experiments employing cell cultures of the respective plant (Wu et al. 2016), growing plants hydroponically in drug-containing growing medium (Madikizela et al. 2018), or by actual field studies whereby plants are cultivated under real farming conditions and irrigated with contaminated water (either prepared in the laboratory or water from an WWTP) (Wu et al. 2014). After harvesting, plants are extracted, and extracts are analyzed commonly by HLPC or GC with mass spectrometric (MS) detection. As for most of the potential metabolites mentioned above standard substances for their unambiguous identification are not available, all information that can be gained from chromatography, and spectrometry should be combined to allow a tentative identification of metabolites with at least acceptable certainty according to the scheme of Schymanski (Schymanski et al. 2014). Besides retention time from chromatography, accurate mass measurement (from high-resolution MS instruments), and MS/MS experiments, the determination of collision cross section (CCS) values via drift-tube ion-mobility mass spectrometry has been added recently as another parameter for the description of potential metabolites detected in the plant extracts (Mlynek et al. 2020).

In this work, we present investigations on the uptake, translocation, and metabolization of four drugs namely amitriptyline (AMT), orphenadrine (ORP), lidocaine (LDO), and tramadol (TRM) using two model plants (cress and pea). Furthermore, to study the kinetics involved in the uptake and translocation of these substances, a set of time-resolved experiments was conducted.

## Experimental

### Chemicals and materials

Amitriptyline hydrochloride (AMT) (Lundbeck AG, Opficon, Switzerland), orphenadrine citrate (ORP) (Meda Pharma GmbH, Vienna, Austria), and tramadol hydrochloride (TRM) (Ratiopharm GmbH, Ulm, Germany) were obtained as pharmaceutical preparation. Lidocaine hydrochloride (LDO) (Pharm.Eur. > 98%) was purchased at a local pharmacy (IRIS Apotheke Kronstorf). Chemical structures are shown in Figure S1 (Supplementary material). An overview of all tablet ingredients can be seen in Table S1 (Supplementary material). Tablets were homogenized with mortar and pestle, and individual stock solutions with a concentration of 1000 mg L^−1^ were prepared in methanol. Further dilutions of lower concentration were prepared by diluting the stock solution with Milli-Q water. For plant treatment, the solutions were further diluted with tap water.

Methanol and acetonitrile were delivered by VWR (Vienna, Austria). Formic acid (eluent additive for LC–MS, 98%) was purchased from Sigma-Aldrich (Steinheim, Germany). Hydrochloric acid 37%, analytical reagent grade, was obtained from Merck (Darmstadt, Germany). Purified water was produced by a Milli-Q water purification system (Millipore, Bedford, MA, USA).

### Plant cultivation and treatment

The cress seeds (*Lepidium sativum* L.) were supplied by Sperli (Everswinkel, Germany), and the pea seeds (*Pisum sativum* L.) from Raiffeisen Ware Austria AG (Austria). All plants were cultivated in tap water without any addition of nutrients. Approximately 5 g of cress seeds were distributed on the grid of the cultivation set, which was subsequently filled with the respective medium (tap water or API solution). One dish carried approximately 300 mL. The API solutions (10 mg L^−1^, 1 mg L^−1^, or 10 μg L^−1^) were prepared by diluting the standard stock solutions with tap water. The plants were then grown on the laboratory bench. Pea seeds were left in darkness on wet paper towels for two days to germinate. This was followed by a growing period of 7 days in a bed of wetted iron-on beads. Afterwards growing was continued in approximately 50 mL of tap water or an API solution. The plants were placed behind a window in the laboratory, and no artificial light cycles were used.

For an investigation regarding uptake, translocation, and possible metabolization of APIs in plants, time study experiments were conducted with cress and peas. The plants were first grown in pure tap water for a week. Subsequently, the pure tap water was replaced by an aqueous solution containing the selected drug, starting the second growing period (16 days). Three replicate samples were taken 1, 2, 4, 8, and 16 days after the start of exposure. Plantlets were rinsed multiple times with distilled water, dried with a paper towel, and stored at − 80 °C until extraction. For each plant species and API, two experiments were conducted. In the first experiment, the plants were exposed to a 1 mg L^−1^ API solution in tap water. During the experiment, repeatedly small volumes of tap water were added to the solution in the cultivation container to compensate for evaporation. This experiment should simulate a single exposition of the drug by a higher concentration of the API. In the second experiment, a 10 µg L^−1^ API solution in tap water was used, which was exchanged with a freshly prepared one at each sampling date. This experiment should represent a continuous supply of the drug as would be the case when irrigating plants with reclaimed water (containing small concentrations of drugs).

### Preparation of plant extracts for tentative identification of metabolites

For setting up an extraction method cress was cultivated in a 10 mg L^−1^ API solution for 7 days. During harvesting, the plants were removed from the cultivation container and separated into roots and leaves. The individual plant materials were then washed thoroughly with tap water and subsequently with deionized water. After drying with kitchen paper, 500 mg ± 1% (fresh plant material) of the individual plant parts were weighed into an Eppendorf tube. Solid–liquid extraction was performed by adding 1 mL of 0.1 M HCl/MeOH (1:1) to the samples. The samples were homogenized using a swing mill (“Star Beater,” VWR, Vienna, Austria) for 15 min at 25 Hz. The homogenized samples were centrifuged for 16 min at 4200 g. The supernatant was attained with a syringe and subsequently filtered into 1.5 mL HPLC glass vials using a 0.45-µm syringe filter (Chromafil PA-45/15 MS, Macherey–Nagel, Düren, Germany). The samples were stored at − 80° C until analysis.

### HPLC / DTIM-QTOF-MS and HPLC / QqQ-MS/MS

The samples were separated by RP-HPLC using an Agilent 1260 HPLC system from Agilent Technologies (Waldbronn, Germany) equipped with a degasser, a quaternary pump, and an autosampler. The separation was performed on a Poroshell 120 EC-C18 column (3 × 75 mm, particle size 2.7 µm, Agilent) that was protected with a C18 guard column (4 × 3 mm, particle size 3 µm) from Phenomenex (Aschaffenburg, Germany).

For HPLC separation, a water/acetonitrile gradient was applied. Starting conditions were set to 95% solvent A (water with 0.1% formic acid) and 5% solvent B (acetonitrile with 0.1% formic acid). From minute 0 to 5, solvent B was increased to 15%; from minute 5 to 10, solvent B was increased to 30%; from minute 10 to 15, solvent B was increased to 50%, followed by 5 min 100% solvent B and 5 min equilibration with starting conditions, resulting in a total run time of 25 min. The flow rate was set to 0.6 mL min^−1^, the temperature of the column heater was 30 °C, and an injection volume of 20 µL was used.

For the tentative identity confirmation of metabolites, the HPLC system was hyphenated with an Agilent 6560 DTIM-QTOF LC–MS/MS (operated either in the “QTOF only” or ion-mobility MS mode) equipped with a Dual AJS ESI source (Agilent Technologies, Waldbronn, Germany).

The DTIM-QTOF-MS was tuned in the “fragile ion” mode and operated in the positive ionization mode with the following source parameters: drying gas temperature 300 °C, drying gas flow rate 10 L min^−1^, nebulizer pressure 50 psi, sheath gas temperature 300 °C, sheath gas flow rate 10 L min^−1^, capillary voltage 3500 V, nozzle voltage 1000 V, and fragmentor 425 V. For MS/MS experiments, nitrogen was used as collision gas, and collision energies of 20 V and 30 V were applied.

The ^DT^CCS_N2_ values were obtained using nitrogen as drift gas and the following parameters for the DTIM device: 4-bit multiplexing, frame rate 0.9 frames s^−1^, IM transient rate 18 transients frame^−1^, max drift time 60 ms, trap fill time 3900 µs, and trap release time 250 µs. Calibration was performed according to a “single-field” approach, allowing the determination of the ^DT^CCS_N2_ values. In order to relate the measured drift times to known and standardized ^DT^CCS_N2_ values of the calibrant analytes, a tune mix calibrant was measured (applying the same conditions as for the samples) before analyzing the actual sample. Drift tube parameters (for “single-field” measurements) were the following: drift tube entrance 1567 V, drift tube exit 217 V, rear funnel entrance 210.5 V, and rear funnel exit 38 V.

### Data processing

For data evaluation, Agilent MassHunter Qualitative Analysis B.07.00, MassHunter Quantitative Analysis B.10.1, MassHunter PCDL Manager B.08.00, PNNL PreProcessor (2020.03.23), and the IM-MS Browser B.10.00 were used.

A database (PCDL) was created, similar as described in a previous study (Mlynek et al. 2020). Therefore, APIs or hydroxylated APIs were in silico combined with common building blocks of phase II and phase III metabolites like glucose or malonic acid in every combination and any number possible. This database (see Table S2, Supplementary material) is used for screening the MS spectra of treated plant samples. The results were verified by a targeted MS/MS experiment, looking at the accurate masses and fragmentation pattern. With the fragmentation pattern, possible sum formulas for metabolites were reconstructed from mass losses.

Ion-mobilty (IM) data files were first demultiplexed using the PNNL PreProcessor software. Afterwards, the data were calibrated with the recorded single-field tune using IM-MS browser. The drift times and the ^DT^CCS_N2_ were determined by first performing a feature extraction (Find features IMFE). Parameters were as follows: processing chromatographic, isotope model common organic molecules, limit charge state z <  = 1, ion intensity >  = 1. The drift times and the collision cross sections of all features were automatically determined by the software. Within all the features found by the software, the analytes of interest were identified according to their m/z values and their retention times.

## Results and discussion

### Studies on the metabolization of the four APIs

It is known from a range of experiments that plants grown hydroponically tend to take up contaminants such as pharmaceuticals and personal care products (PPCPs) from the growing solution (for exemplary references see Madikizela et al. 2018). Starting point for this work was the analysis of common water hyacinth (*Eichhornia crassipes* Mart.) samples collected by researchers in two South African rivers (Mbokodweni River and Mdloti River). Extracts from these plants contained seven APIs, namely citalopram, telmisartan, amitriptyline (AMT), orphenadrine (ORP), tramadol (TRM), atenolol, and lidocaine (LDO). To study uptake, translocation, and metabolization of these compounds, two model plants (cress and pea) were selected, and a range of experiments was designed. As investigations on citalopram, atenolol (Reichl et al. 2018), and telmisartan (Lang et al. 2021) had already been published by our group, further work was restricted to the remaining four APIs (AMT, LDO, ORP, and TRM). The protocol for the preparation of extracts from fresh plant material was based on experience from research conducted in our lab throughout the last years (Emhofer et al. 2018, 2019; Reichl et al. 2018) (for details, see the “Preparation of plant extracts for tentative identification of metabolites” section). For these studies, cress was cultivated in tap water containing the respective API at a level of 1 mg L^−1^. After 21 days, the plant material was harvested, dried, roots and upper part (leaves) of the plant were separated, and extracts were prepared employing the extraction protocols described in the Experimental section.

Subsequently, three sets of experiments each (differing in the concentration of the APIs added to the growing solution) were prepared for cultivating cress and pea. Two of them namely the plants grown in solutions containing 10 mg L^−1^ and 1 mg L^−1^ of the selected pharmaceutical were employed to study the uptake and translocation of the parent drugs and to search for potential metabolites formed within the plant (the third, containing 0.01 mg L^−1^ was employed for the time studies described in the “Time studies on the uptake and translocation of four APIs by cress and pea” section). To differentiate between metabolites already formed within the growing solution and those actually formed in the plant and to identify drug degradation due to hydrolysis or UV-light, comparative analysis of a “window solution,” i.e., a solution of the respective substance objected to the same environmental conditions (including sunlight) as the plants was performed. Thereby no changes in the concentration of the APIs and no degradation products were detected over the timespan of the experiment.

In a first experiment, designed to search for potential drug-related metabolites, all four parent drugs (AMT, LDO, ORP, and TRM) were detected in roots, leaves, and stem of the model plants after exposure for 7, 14, and 21 days. Furthermore, a number of primary metabolites listed in Table 1 could be tentatively identified. Information extracted from the HPLC / DTIM-QTOF-MS/MS runs leading to the proposal of tentative structures included the following: some rough estimation of polarity (by comparing retention times on RP-columns), accurate mass (allowing proposing molecular formulas), and MS/MS spectra recorded at different collision energies (providing some structural information). Additionally, the use of an MS instrument equipped with a drift-tube ion-mobility unit allowed the determination of ^DT^CCS_N2_, giving some idea about similarities and dissimilarities in the three-dimensional structure of the analytes. AMT underwent N-demethylation and hydroxylation. LDO showed N-deethylation also combined with hydroxylation as well as degradation to 2,6 xylidine whereby the latter substance was further modified by hydroxylation or oxidation of one methyl group to the corresponding benzoic acid. For ORP, only two primary metabolites namely N-demethyl-orphenadrine and N,N-didemethyl-orphenadrine were found. Finally, for TRM several phase I metabolites were formed either by N-, and O-demethylation or by hydroxylation. These transformation products are also known from metabolism in humans. Subsequently, LC–MS runs were checked via an in silico database (see Table S2, Supplementary material), previously developed in our lab (Mlynek et al. 2021), to search for potential phase II and phase III metabolites. Surprisingly, no secondary metabolites were detected for LDO and TRM, and only glycosylated species were found for the other two compounds.

**Table 1:** Data of four APIs and tentatively identified metabolites. If no CCS values are provided, the ion abundance was too low for accurate CCS determination

Name	Formula	RT [min]	m/z [M + H]^+^ calc	m/z [M + H]^+^ meas	Error [ppm]	CCS (Å^2^)	MS/MS fragments [m/z]	confirmation criteria level ^a^
*Amitriptyline*								
AMT	C_20_H_23_N	13.7	278.1903	278.1909	2.2	167.6	233.13, 191.09, 91.05	1
AMT-OH	C_20_H_23_NO	10.0	294.1852	294.1851	-0.3	168.6	276.17, 231.12, 191.09	3
NTP	C_19_H_21_N	16.6	264.1747	264.1753	2.3	165.8	233.13, 219.17, 91.05	3
NTP-OH	C_19_H_21_NO	9.8	280.1696	280.1698	0.7	166.5	262.16, 231.12	3
DMN	C_18_H_19_N	13.4	250.159	250.1595	2.0	165.1	233.13, 191.09, 91.05	3
AMT-Glc	C_26_H_33_NO_5_	12.5	440.2431	440.2438	1.6	201.5	278.19, 233.13	3
AMT-OH-Glc	C_26_H_33_NO_6_	8.9	456.2381	456.2386	1.1	205.1	294.18, 276.17	3
*Lidocaine*								
LDO	C_14_H_22_N_2_O	6.0	235.1805	235.1809	1.7	156.6	86.10	1
LDO-OH	C_14_H_22_N_2_O_2_	3.1	251.1754	251.1751	-1.2	*	86.10	3
MEGX	C_12_H_18_N_2_O	4.7	207.1492	207.1486	-2.9	*	58.07	3
*Orphenadrine*								
ORP	C_18_H_23_NO	13.0	270.1852	270.1856	1.5	166.4	181.10, 166.08	1
ORP-Glc	C_24_H_33_NO_6_	12.0	432.2381	432.2383	0.5	199.8	270.18, 181.10	3
*Tramadol*								
TRM	C_16_H_25_NO_2_	8.2	264.1958	264.1950	-3.0	160.7	58.07	1
ODT	C_15_H_23_NO_2_	5.4	250.1802	250.1806	1.6	159.7	58.07	3
NDT	C_15_H_23_NO_2_	8.4	250.1802	250.1800	-0.8	156.7	44.05	3
TRM-OH	C_16_H_25_NO_3_	8.9	280.1907	280.1912	1.8	166.6	262.18, 58.07	3

^*^Concentrations too low for CCS determination

^a^Schymanski et al., Environ. Sci. Technol. 2014, 48, 2097 − 2098

### Time studies on the uptake and translocation of four APIs by cress and pea

**Fig. 1:**
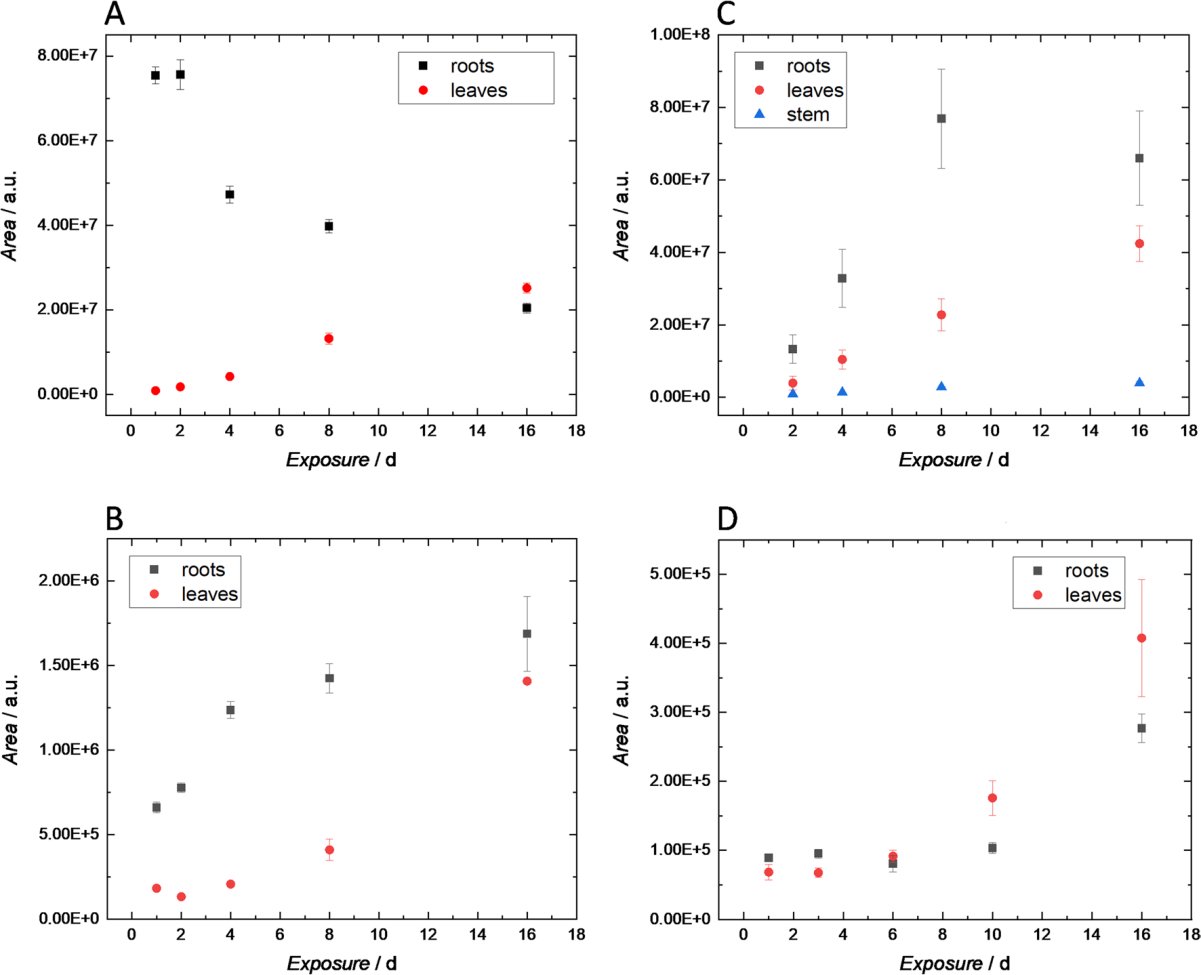
TRM time study in cress (**A**) and (**B**) and pea (**C**) and (**D**). Data points refer to peak areas (mean values from three replicates) obtained for TRM in extracts from roots, stem, and leaves. Plants were cultivated for 16 days in solutions containing TRM at a level of 1 mg L^−1^ (**A**) and (**C**) and 10 µg L^−1^ (**B**) and (**D**). Error bars represent standard deviations

Two different types of time studies investigating the uptake and translocation of AMT, LDO, ORP, and TRM by cress and pea were conducted. In one case, plantlets were grown in water spiked with the corresponding drug at a level of 1 mg L^−1^. Whenever samples were taken (after 1, 2, 4, 8, and 16 days), the lowered water level (due to evaporation) was adjusted by the addition of pure water. For the second type, plants were grown in an aqueous solution containing 0.010 mg L^−1^ of the respective pharmaceutical. Whenever plants were removed for sampling, the complete solution was renewed. The first setup should mimic a situation where plants get in contact with water that is severely contaminated with drug residues; approach two resembled the scenario when for example plants are regularly irrigated with treated wastewater still containing the investigated drug residues at trace levels. Thereby, two different trends could be observed. When plants were subjected to the higher concentration of the drug (1 mg L^−1^) for cress, maximum peak areas for root extracts were already obtained after 1 day of exposure. Subsequently, peak areas for the parent drug in roots decreased steadily, whereas those in leaves increased, showing the subsequent transport of the substances within the plant. For pea, concentration in the roots increased till day eight—indicating a slower take up of the parent drug compared to cress. After day eight, also in this plant, a decrease in the drug concentrations in root extracts was observed. In leaves and stem of pea, peak areas (similar as in cress) showed a steady increase till the end of the experiment, hinting to the transport of the parent drug from the roots to the upper plant parts. When the second approach was used (solution containing 0.010 mg L^−1^ that was regularly replaced by a fresh one), peak areas for roots showed a steady increase from day one. This can be understood as evidence that the kinetics of drug uptake is substantially influenced by the availability of higher amounts of the drug in the growing solution. Concurrently, an increase in the leaves was observed. Figure 1 shows these trends on the example of tramadol in cress and pea. Trends for the other substances can be seen in Figs. S2–S23. Not for all substances, clear trends could be observed. This might be attributed to biological variations but also to low concentrations, that in some cases led to increased erros in the determination of peak areas.

**Fig. 2:**
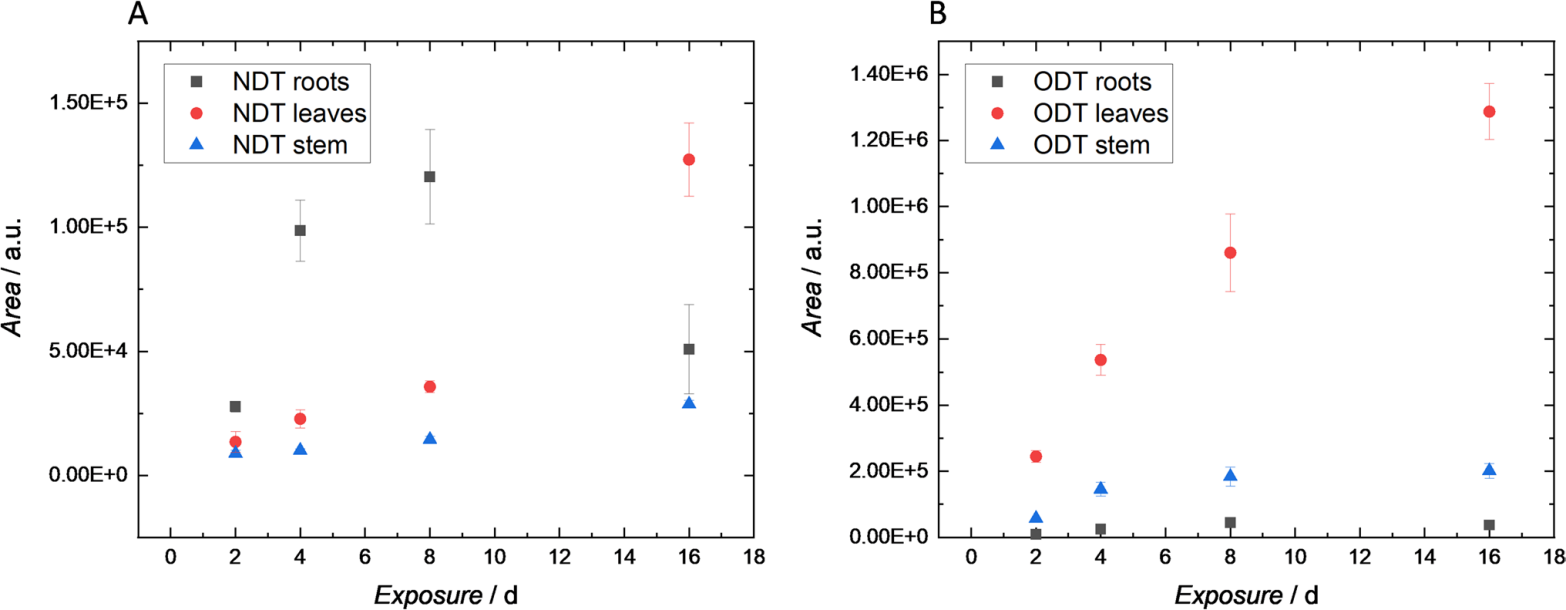
Time study of TRM metabolites NDT (**A**) and ODT (**B**) in peas after exposure to a 1 mg L^−1^ TRM solution. Data points refer to peak areas (mean values from three replicates). Error bars represent standard deviations

In addition, also some of the phase I metabolites, originally detected in plants when a much higher concentration of the parent drug was applied (10 mg L^−1^), could be found during these time studies. Focusing on the experiments conducted with the 1 mg L^−1^ solutions, four AMT metabolites (AMT-OH, NTP, NTP-OH, and DMN) were found in cress roots and leaves. For pea, a similar result was obtained, but with much slower kinetics, meaning that only after day eight the metabolites were detected in roots, stem, and leaves. A more detailed picture of this behavior can be seen from Table 2. Switching to LDO and TRM, LDO-OH and MEGX (which metabolites originating from LDO) were found in cress and pea. In the case of TRM, ODT and NDT could be identified. In most cases, a gradual increase of metabolite concentrations over time could be detected. As a typical example, the time profile of NDT and ODT in pea is presented in Fig. 2. Here, substantially larger peak areas (factor of ten) were obtained for ODT. Whereas this metabolite showed a steady increase over time with largest peak areas obtained for leaf extracts, NDT showed an increase in the roots till day eight followed by a significant decrease from this time on. Simultaneously, the concentration in leaves showed a steep increase. Extracts from pea stem exhibited only marginally higher peak areas at the end of the observation period.

**Table 2:** Metabolites of AMT detected in different plant parts of cress and pea exposed to a 1 mg L^−1^ solution over a period of 16 days. Values refer to average peak areas from measurements in triplicate

Metabolite	Day 1			Day 2			Day 4			Day 8			Day 16		
Cress															
	Roots		Leaves	Roots		Leaves	Roots		Leaves	Roots		Leaves	Roots		Leaves
AMT-OH*	3.7		0.5	5.6		1.5	3.7		2.0	3.7		5.5	2.0		11
NTP**	7.5		1.2	5.2		4.8	3.0		5.0	1.7		11	2.0		16
NTP-OH**	1.7		n.d	2.7		0.5	4.7		0.8	3.0		4.5	5.2		13
DMN***	7.8		1.1	3.5		3.1	1.7		3.4	1.5		7.0	1.7		6.3
Pea															
	Roots	Stem	Leaves	Roots	Stem	Leaves	Roots	Stem	Leaves	Roots	Stem	Leaves	Roots	Stem	Leaves
AMT-OH*				6.0	0.8	0.8	2.4	0.8	0.8	3.2	2.0	8.0	4.0	2.0	11
NTP*				3.2	0.3	0.1	3.2	0.6	0.2	7.2	1.3	2.7	8.5	3.1	4.5
NTP-OH**				0.7	n.d	n.d	0.7	n.d	n.d	1.0	1.0	2.8	1.7	1.2	4.5
DMN***				1.8	n.d	n.d	1.1	n.d	n.d	1.8	n.d	2.0	1.8	1.0	5.7

*n.d.* not detected, *area [arbitrary units] × 10^6^, **area [arbitrary units] × 10^5^, ***area [arbitrary units] × 10^4^

## Conclusions

Pharmaceuticals may be taken up and metabolized by plants if grown in drug-containing solutions. In this work, we were able to demonstrate on the example of cress and pea (used as model plants) that four drugs, namely AMT, LDO, ORP, and TRM are taken up, translocated, and bio-transformed into a series of metabolites. The selection of the drugs used in this study was based on the analysis of hyacinth samples collected from South African rivers. Time studies revealed that the abundance of the parent drug and its metabolites varies over time, whereby changes in the distribution of both the parent drug and the formed metabolites between the root, the stem (for pea only), and the leaves of the plant also showed variations over the growing period. The use of a DTIM-QTOF-MS allowed the determination of the ^DT^CCS_N2_ as an additional specific parameter for each analyte.

### Supplementary Information

Below is the link to the electronic supplementary material.

Supplementary file1 (DOCX 1286 kb)

## Data Availability

All data and materials are included in this published article.
