# Supplementary material for: Uptake, translocation, and metabolization of amitriptyline, lidocaine, orphenadrine, and tramadol by cress and pea
Source: Environ Sci Pollut Res Int. 2024 Feb 16;31(13):19649–57. doi: 10.1007/s11356-024-32379-x (PMC10927770; doi:10.1007/s11356-024-32379-x)
Supplement: Supplementary file 1 — Supplementary file1 (DOCX 1286 kb) [file 11356_2024_32379_MOESM1_ESM.docx]

**UPTAKE, TRANSLOCATION AND METABOLIZATION OF AMITRIPTYLINE, LIDOCAINE, ORPHENADRINE AND TRAMADOL BY CRESS AND PEA**

Anna Detzlhofer^1^, Christian Grechhamer^1^, Lawrence Madikizela^2^, Markus Himmelsbach^1^, Franz Mlynek^1^, Wolfgang Buchberger^1^ and Christian W. Klampfl^1^*

^1^ Institute of Analytical and General Chemistry, Johannes Kepler University, Altenberger Strasse 69, 4040 Linz, Austria

^2^ Institute for Nanotechnology and Water Sustainability, College of Science, Engineering and Technology, University of South Africa, Florida Science Campus, 1710, South Africa

**SUPPLEMENTARY MATERIAL**


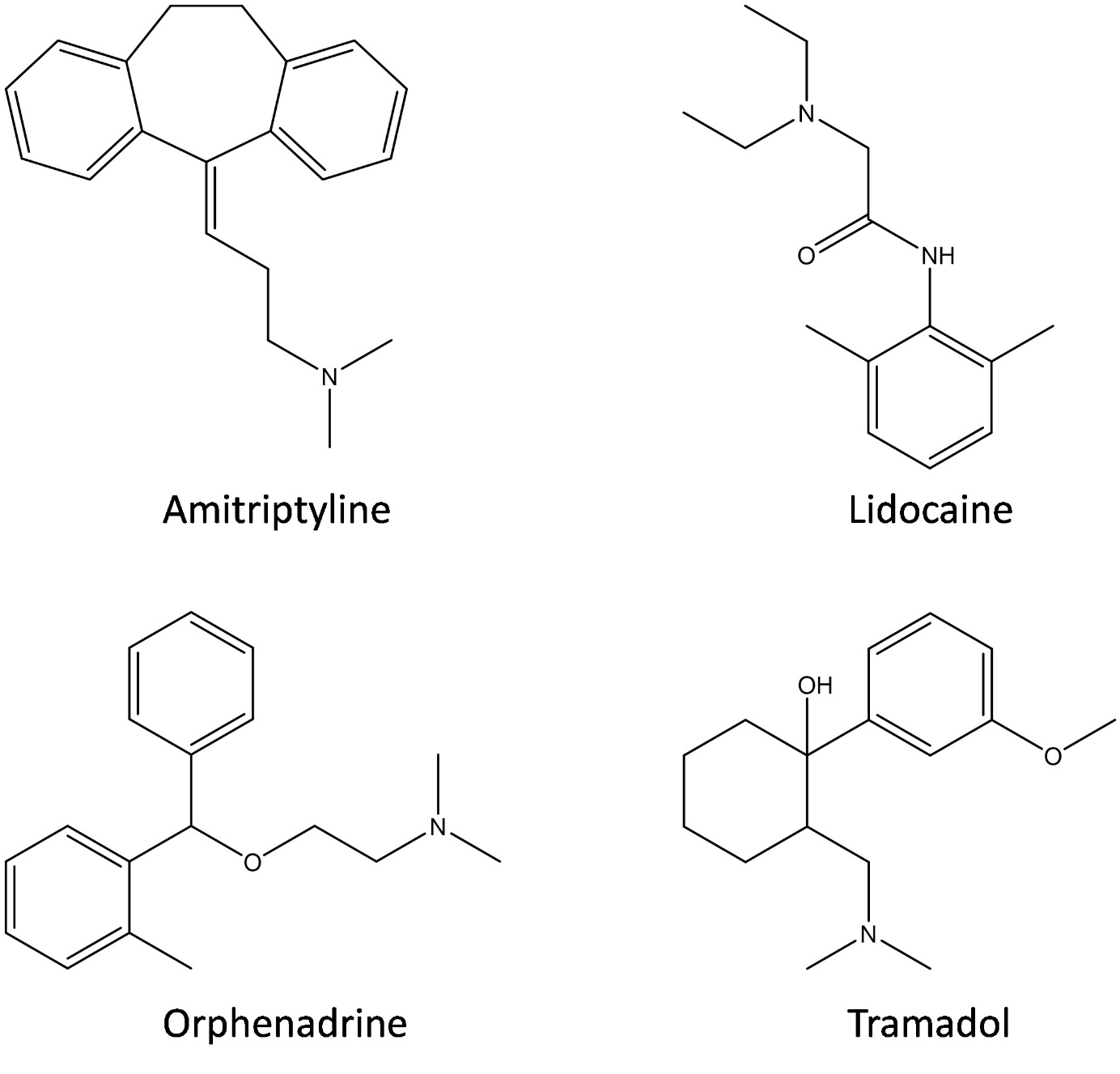


Figure S1: Structures of the investigated API´s


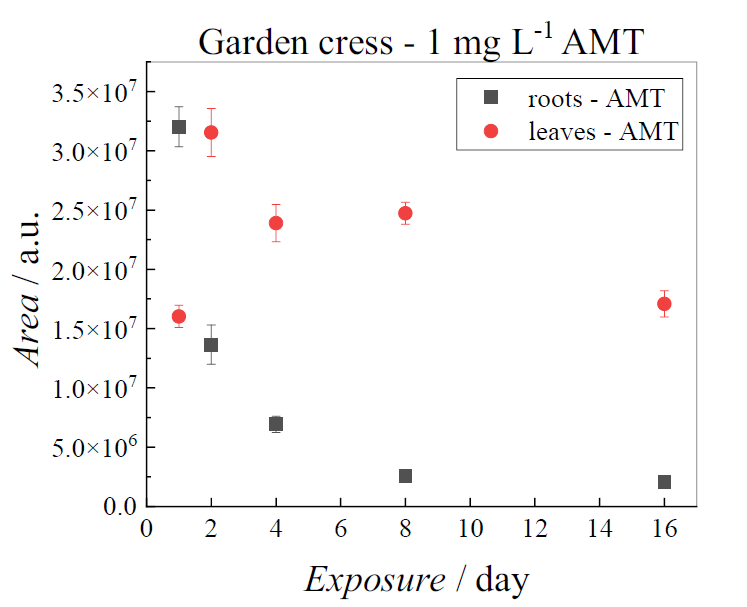


Figure S2: AMT 1 mg L^-1^ time study in cress for 16 days.


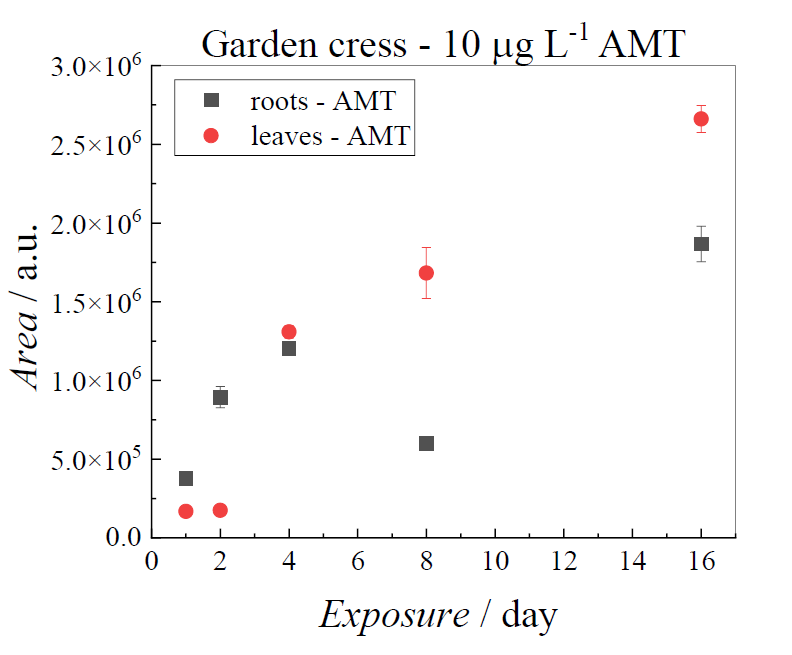


Figure S3: AMT 10 µg L^-1^ time study in cress for 16 days.


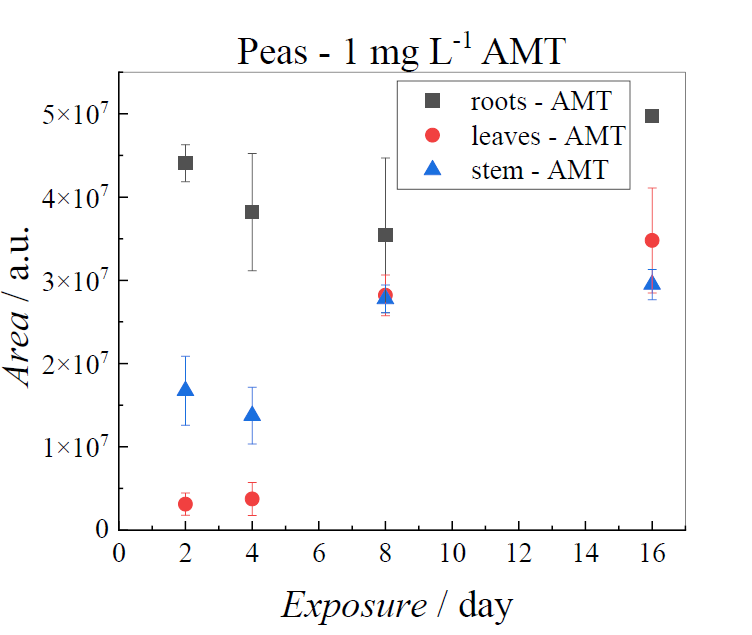


Figure S4: AMT 1 mg L^-1^ time study in pea for 16 days.


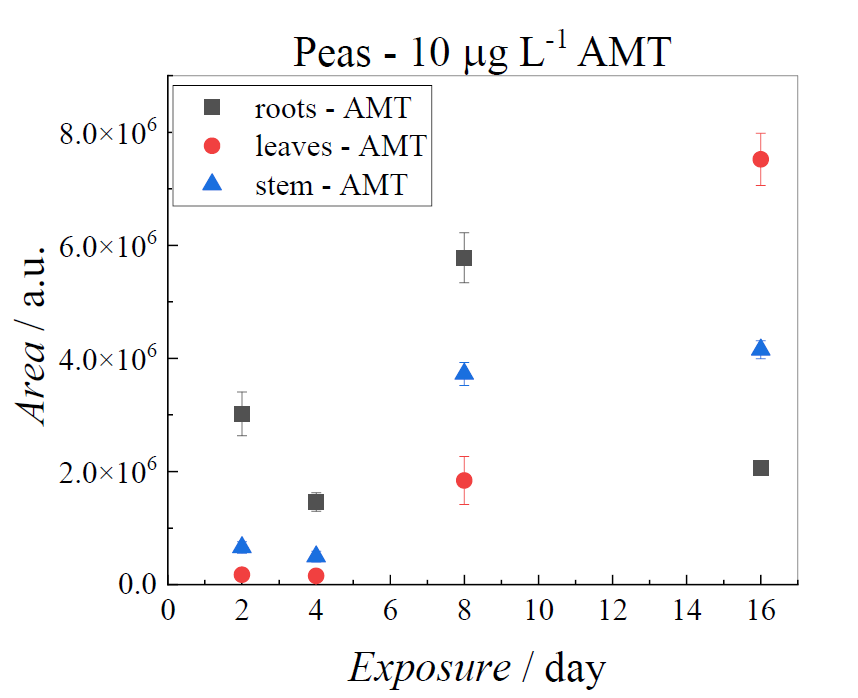


Figure S5: AMT 10 µg L^-1^ time study in pea for 16 days.


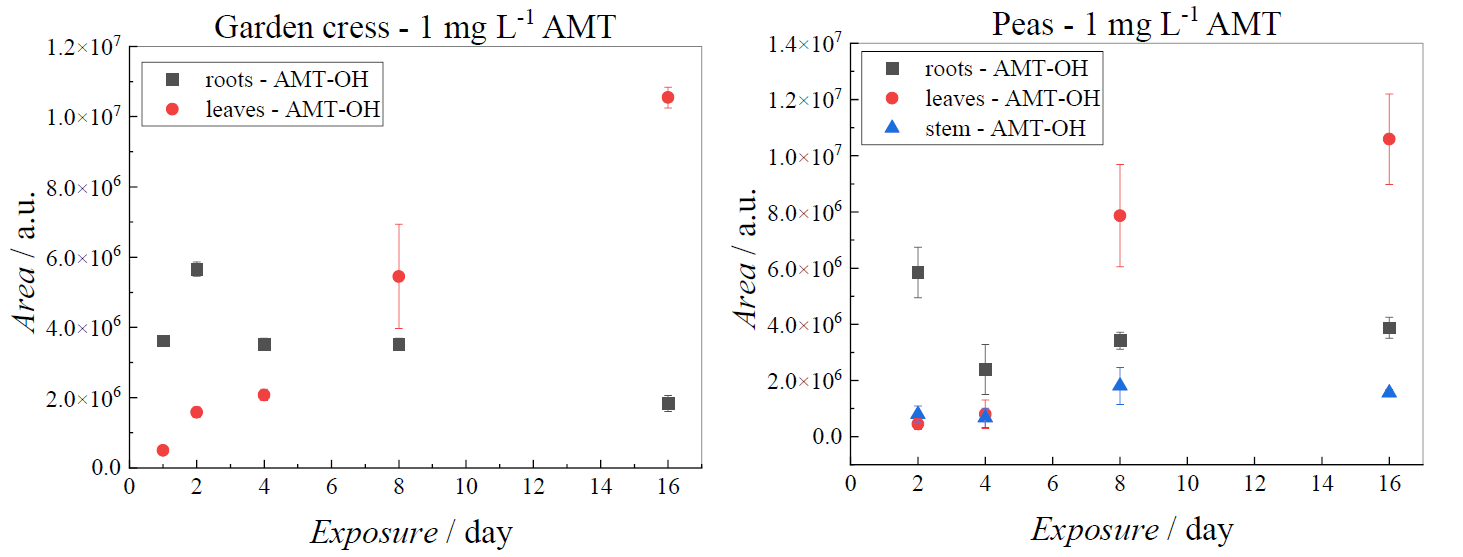


Figure S6: Time study of AMT-OH in cress (left) and pea (right) exposed to a 1 mg L^-1^ AMT solution for 16 days.


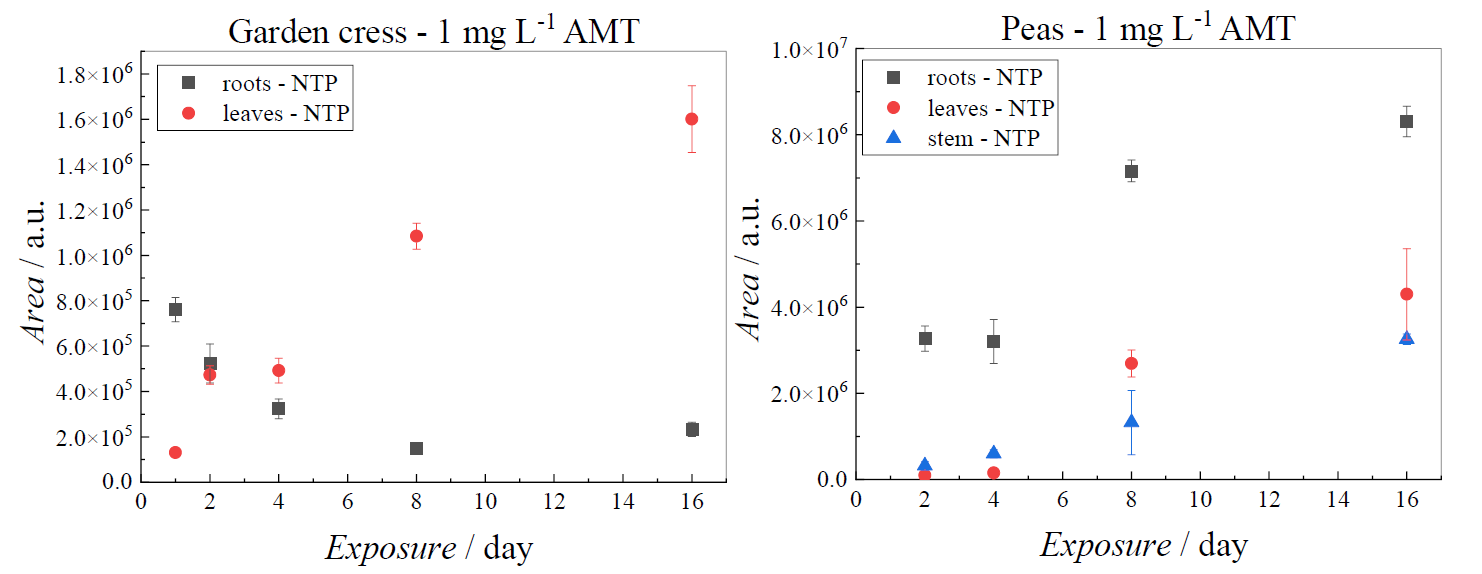


Figure S7: Time study of NTP in cress (left) and pea (right) exposed to a 1 mg L^-1^ AMT solution for 16 days.


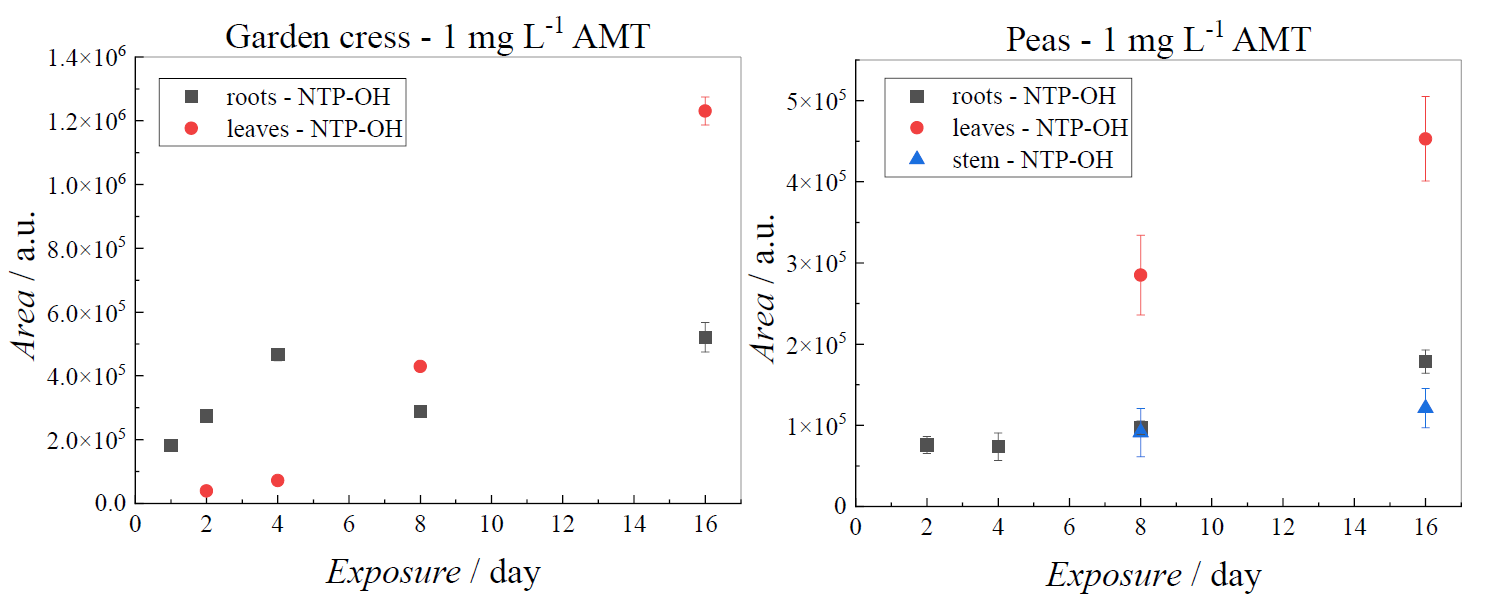


Figure S8: Time study of NTP-OH in cress (left) and pea (right) exposed to a 1 mg L^-1^ AMT solution for 16 days.


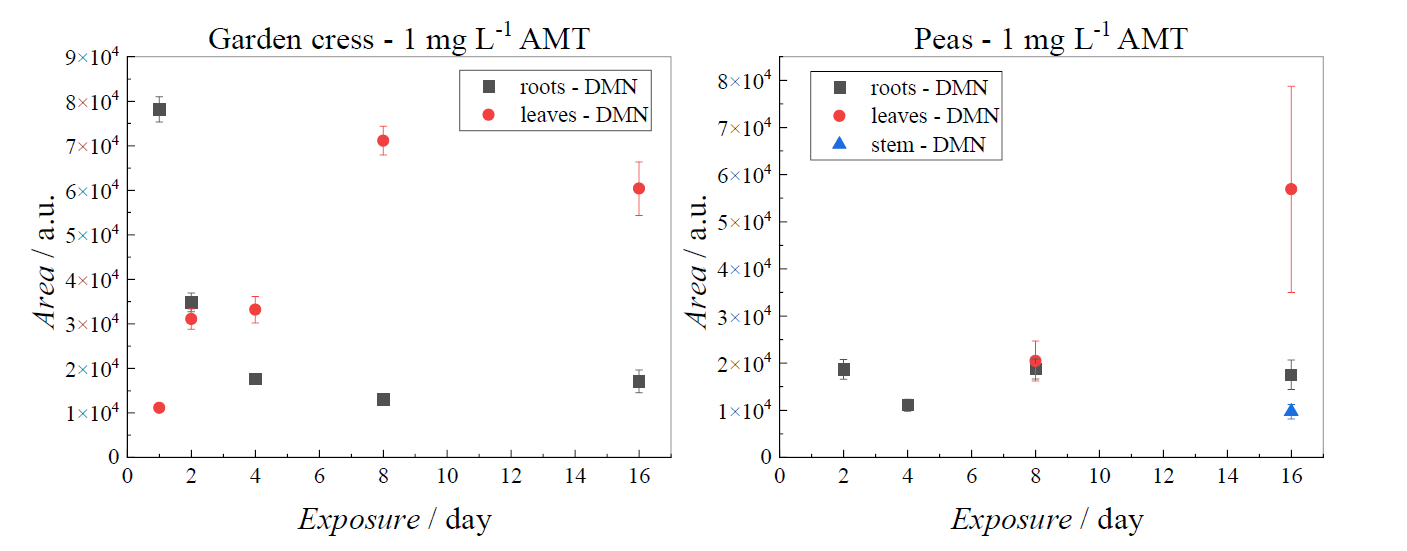


Figure S9: Time study of DMN in cress (left) and pea (right) exposed to a 1 mg L^-1^ AMT solution for 16 days.


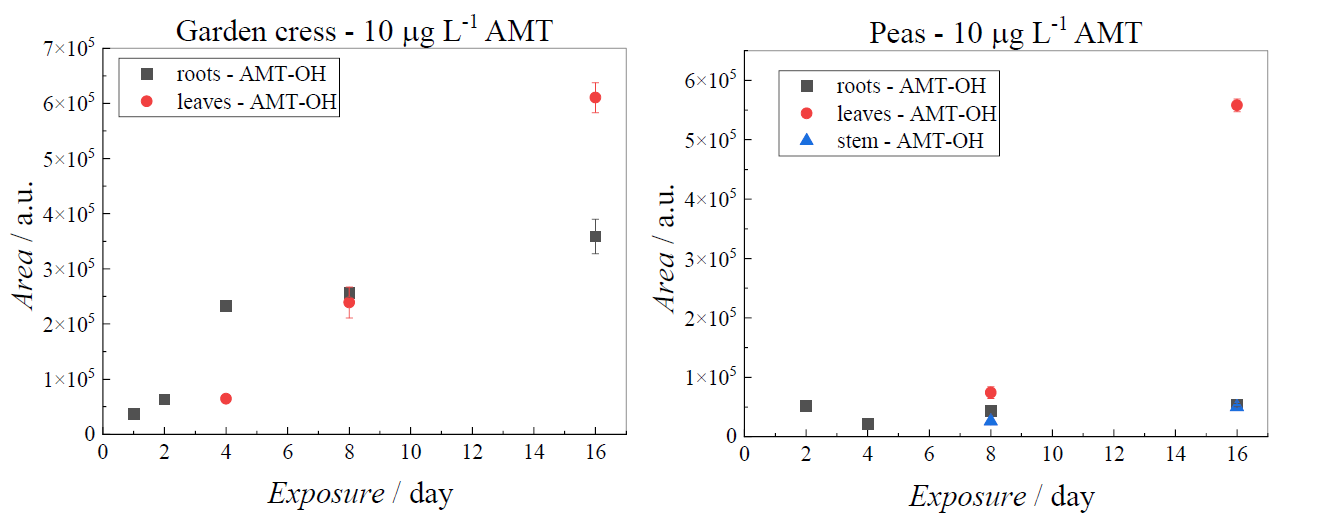


Figure S10: Time study of AMT-OH in cress (left) and pea (right) exposed to a 10 μg L^-1^ AMT solution for 16 days.


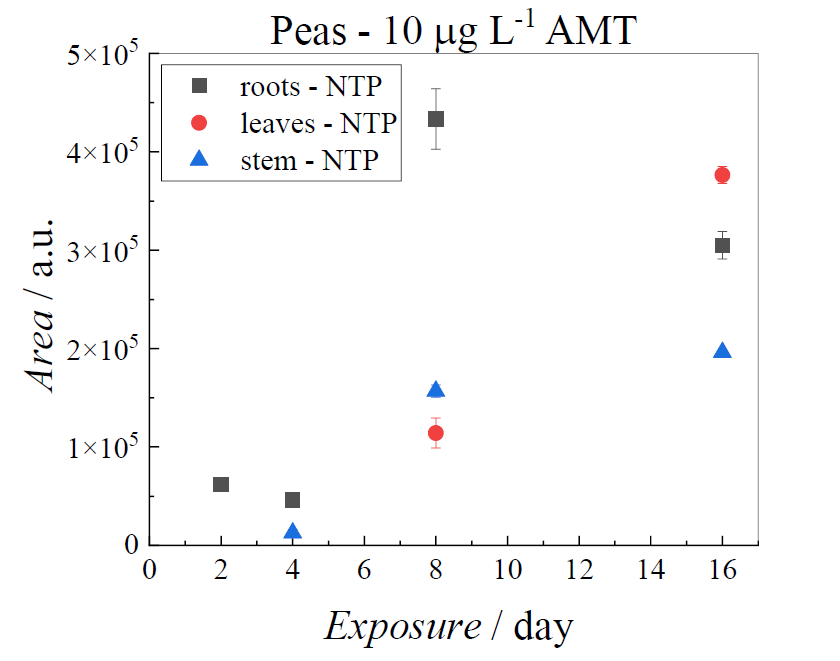


Figure S11: Time study of NTP in pea exposed to a 10 μg L^-1^ AMT solution for 16 days.


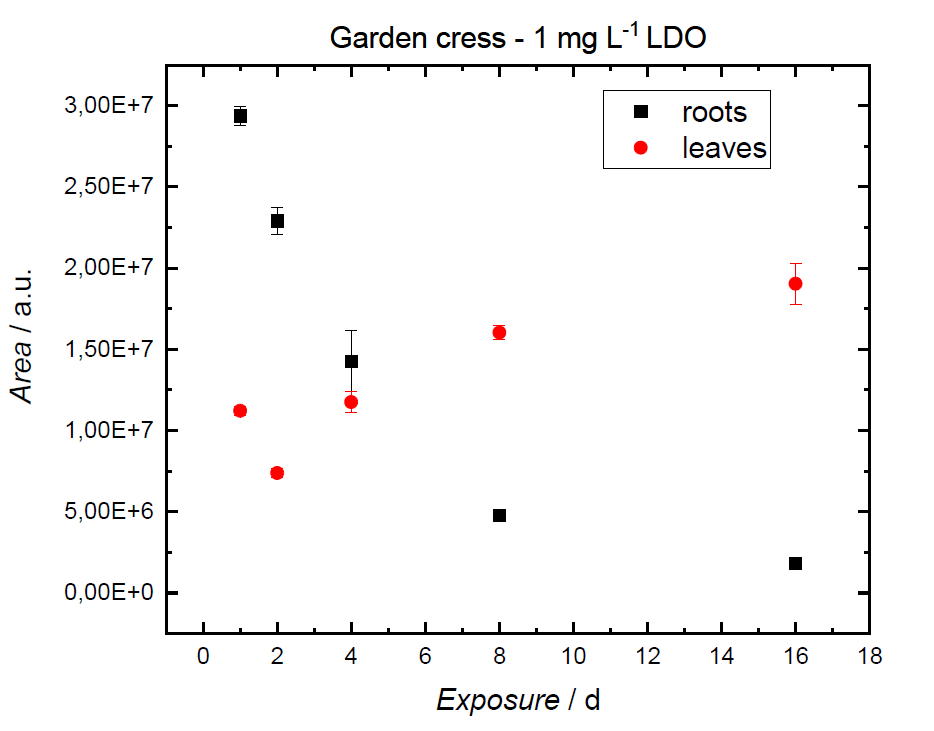


Figure S12: LDO 1 mg L^-1^ time study in cress for 16 days.


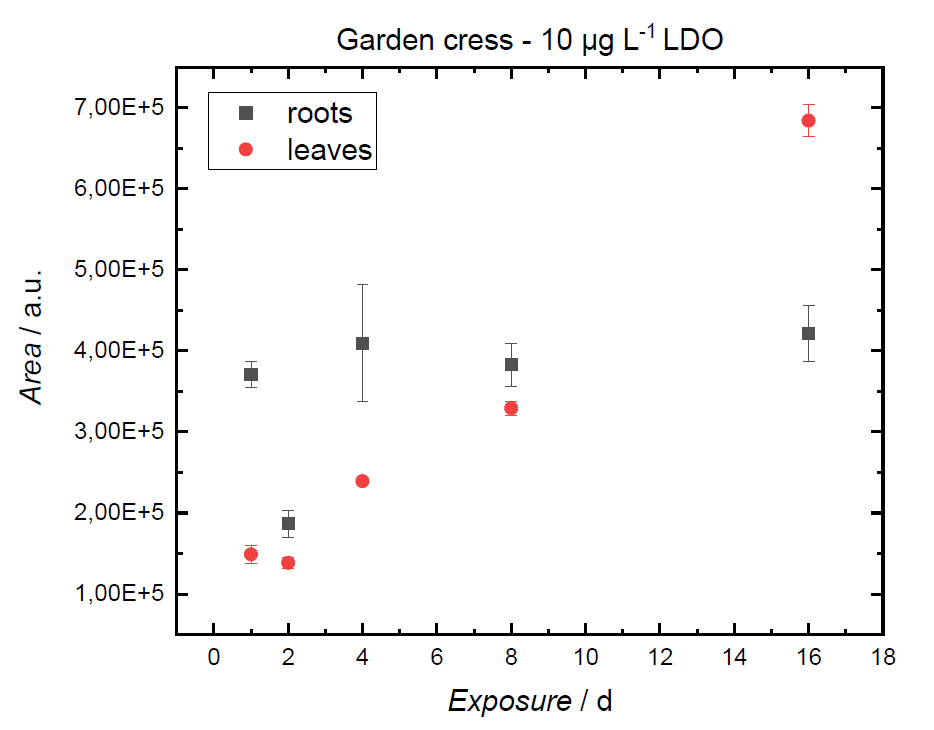


Figure S13: LDO 10 µg L^-1^ time study in cress for 16 days.


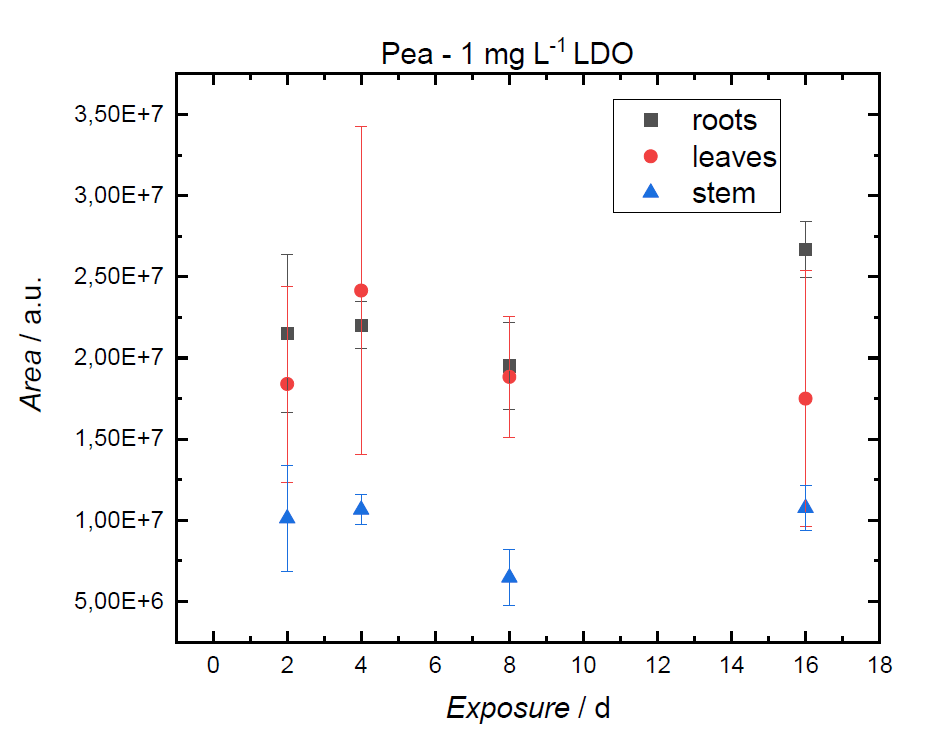


Figure S14: LDO 1 mg L^-1^ time study in pea for 16 days.


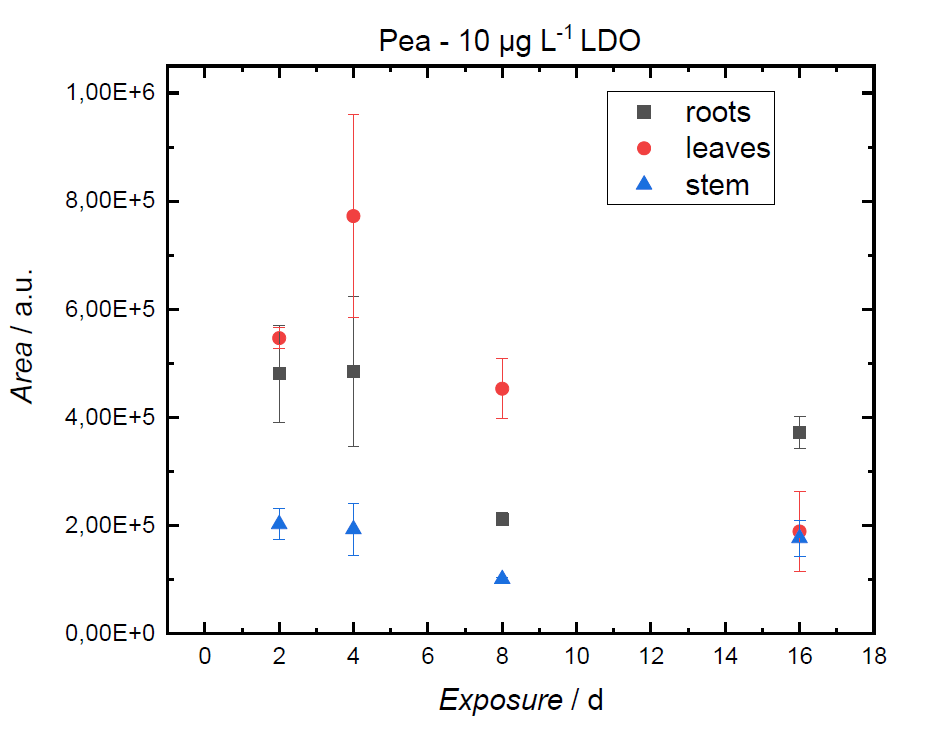


Figure S15: LDO 10 µg L^-1^ time study in pea for 16 days.


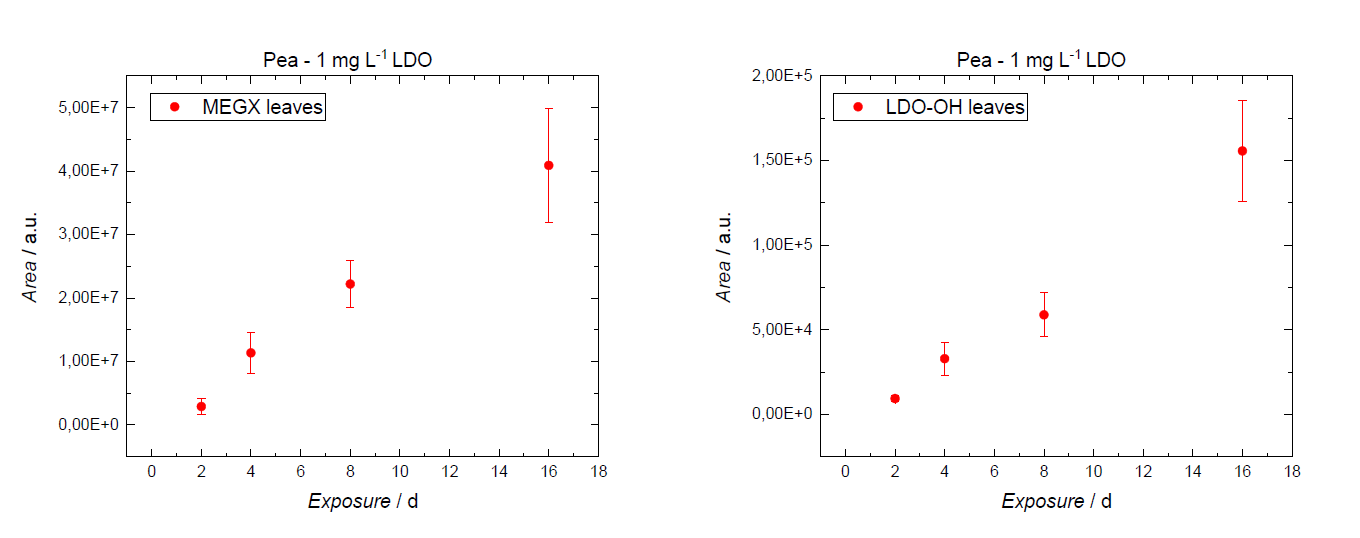


Figure S16: Time-study of MEGX (left) and LDO-OH (right) in pea exposed to a 1 mg L^-1^ LDO solution for 16 days.


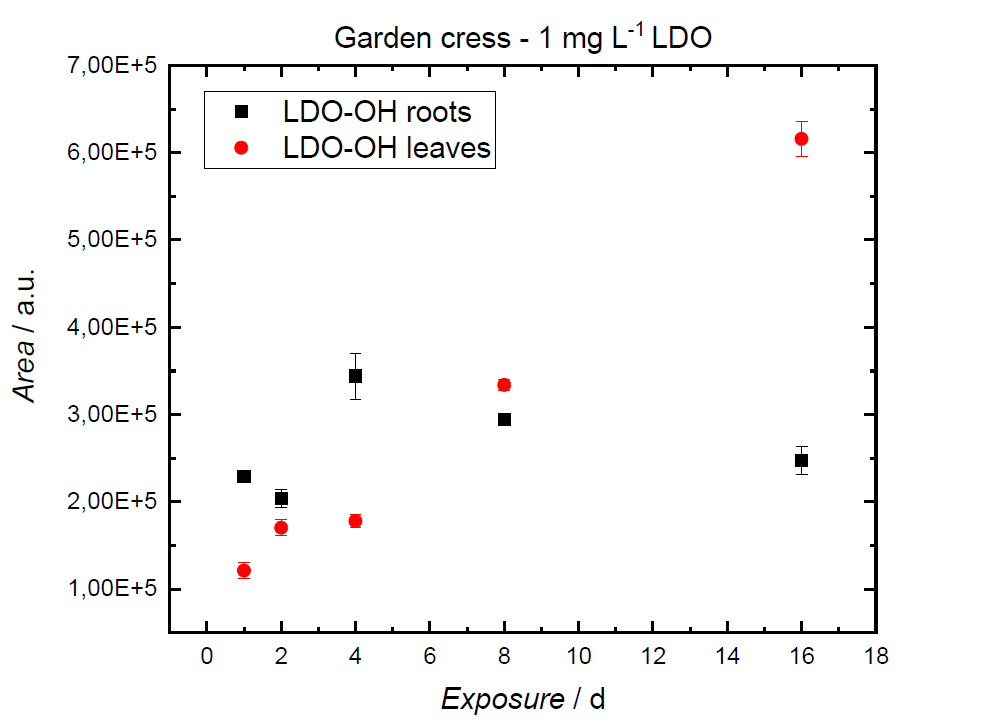


Figure S17: Time-study of LDO-OH in cress exposed to a 1 mg L^-1^ LDO solution for 16 days.


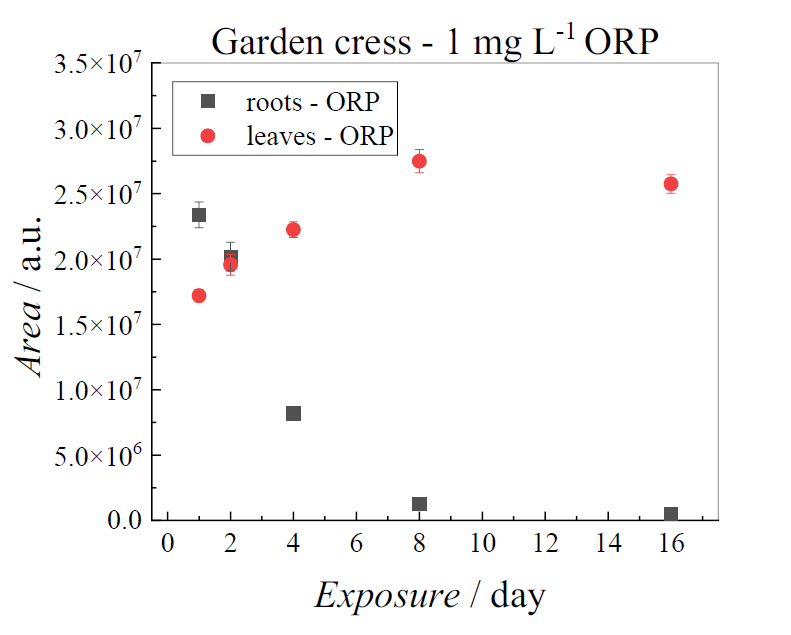


Figure S18: ORP 1 mg L^-1^ time study in cress for 16 days.


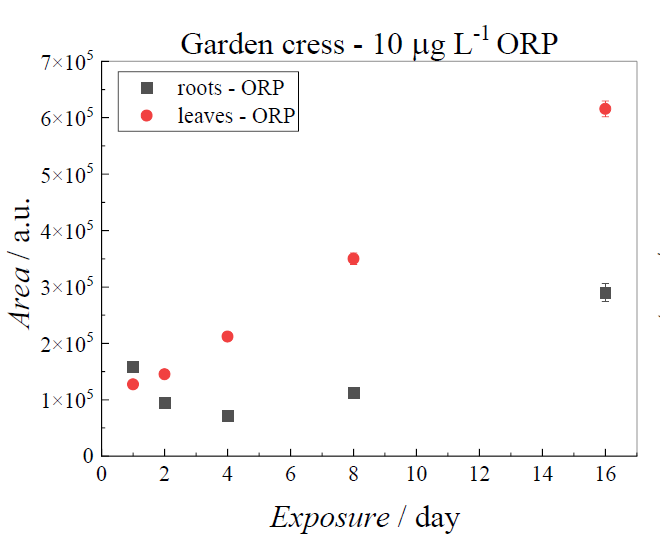


Figure S19: ORP 10 µg L^-1^ time study in cress for 16 days.


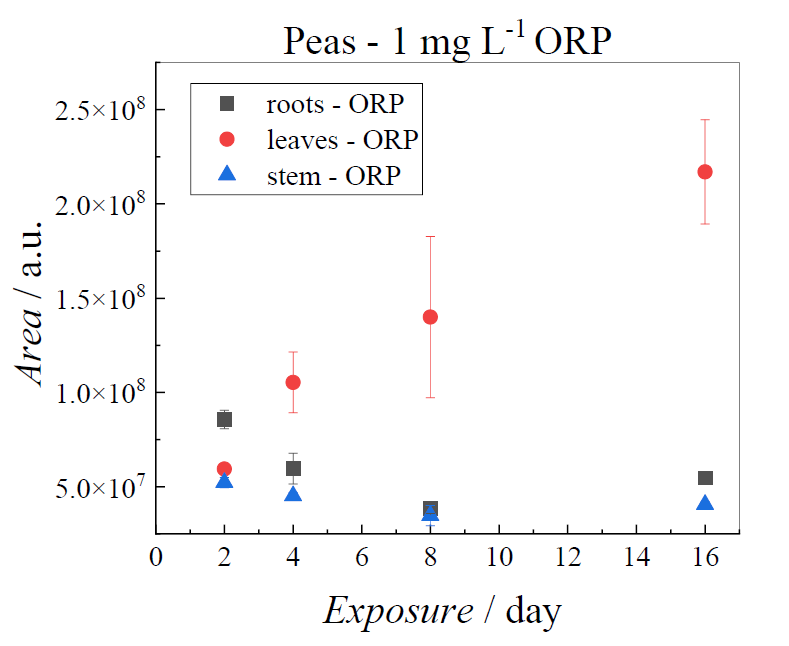


Figure S20: ORP 1 mg L^-1^ time study in pea for 16 days.


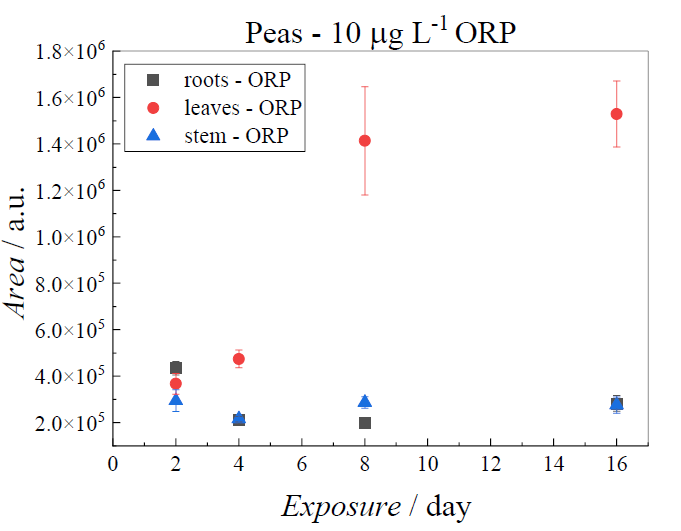


Figure S21: ORP 10 µg L^-1^ time study in pea for 16 days.


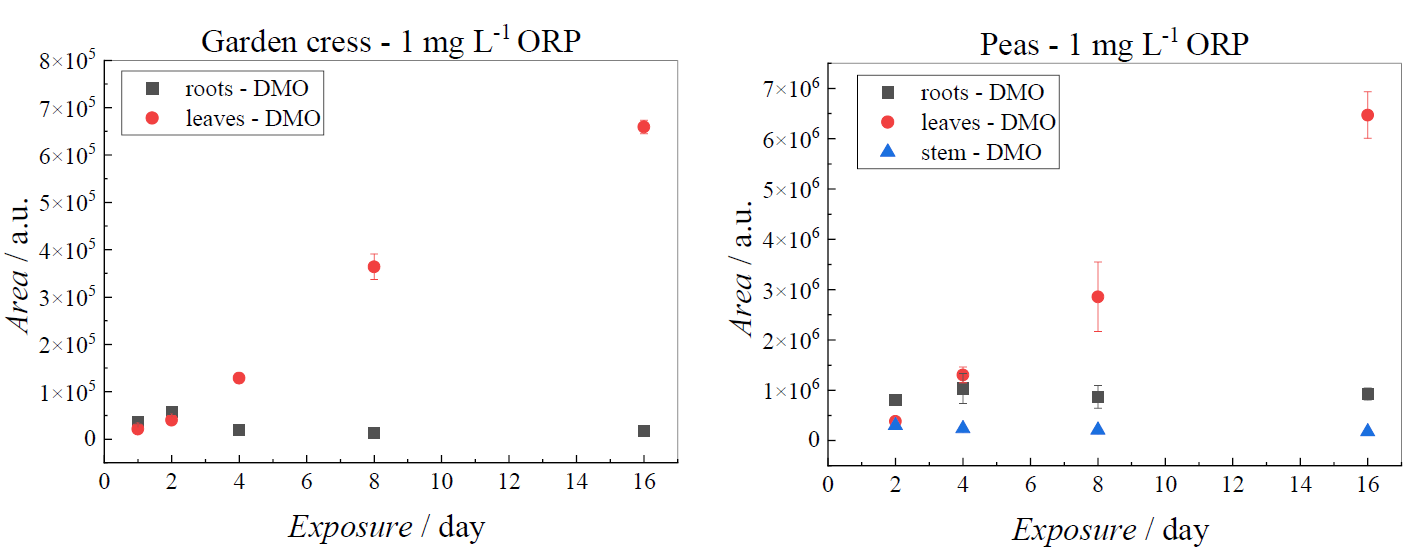


Figure S22: Time study of DMO in cress (left) and pea (right) exposed to a 1 mg L^-1^ ORP solution for 16 days.


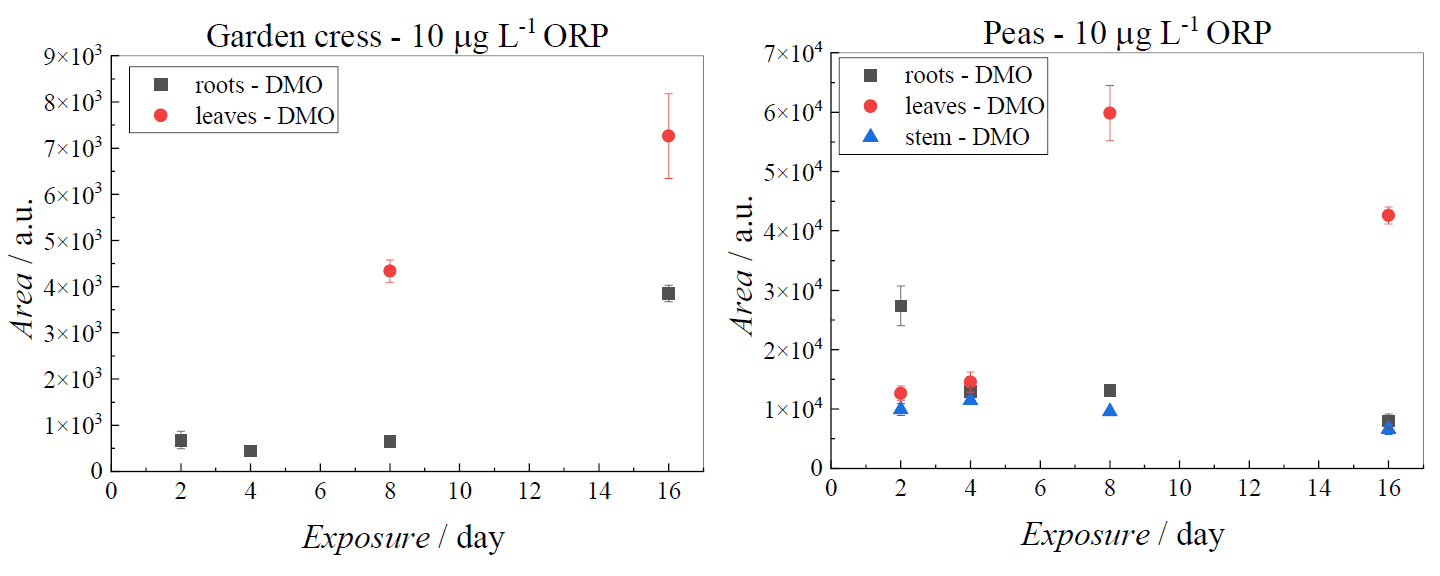


Figure S23: Time study of DMO in cress (left) and pea (right) exposed to a 10 μg L^-1^ ORP solution for 16 days.

Table S1: Pharmaceutical preparations

| Drug Name | Active ingredient | Dosage | Excipients acording to package insert | Manufacturer |
| --- | --- | --- | --- | --- |
| Saroten | Amitriptyline Hydrochloride | 10 mg | corn starch, lactose monohydrate, colloid-hydrogenated silicon, microcrystalline Cellulose, Crospovidone, Croscarmellose Sodium, Magnesium Stearate, Macrogol 400 Dye: Opadry-OY-S-9470 red-brown (E 172; E 171) | Lundbeck AG, Opficon, Switzerland |
| Norgesic | Orphenadrine citrate  Paracetamol | 35 mg  450 mg | Microcrystalline cellulose, pregelatinized starch, Magnesium stearate, colloidal silicon dioxide, gelatin. | Meda Pharma GmbH, Vienna, Austria |
| Tramadol | Tramadol Hydrochloride | 50 mg | Microcrystalline cellulose, hypromellose, magnesium stearate (Ph.Eur.). | Ratiopharm GmbH, Ulm, Germany |

Table S2: List of conjugates for phase II metabolization of xenobiotics (X) in plants.

| **Compound Abbreviation** | **Conjugate sum formula** | **Conjugate exact mass** |
| --- | --- | --- |
| X-Glc | C6H10O5 | 162.0528 |
| X-Glc-Glc | C12H20O10 | 324.1056 |
| X-Glc-Glc-Glc | C18H30O15 | 486.1585 |
| X-Glc-Glc-Glc-Glc | C24H40O20 | 648.2113 |
| X-Glc-Mal | C9H12O8 | 248.0532 |
| X-Glc-Glc-Mal | C15H22O13 | 410.1060 |
| X-Glc-Glc-Glc-Mal | C21H32O18 | 572.1589 |
| X-Glc-Glc-Glc-Glc-Mal | C27H42O23 | 734.2117 |
| X-Glc-Mal Methylester | C10H14O8 | 262.0689 |
| X-Glc-Glc-Mal Methylester | C16H24O13 | 424.1217 |
| X-Glc-Glc-Glc-Mal Methylester | C22H34O18 | 586.1745 |
| X-Glc-Glc-Glc-Glc-Mal Methylester | C28H44O23 | 748.2273 |
| X-Mal | C3H2O3 | 86.0004 |
| X-Mal-Mal | C6H4O6 | 172.0008 |
| X-Mal-Mal-Mal | C9H6O9 | 258.0012 |
| X-Mal Methylester | C4H4O3 | 100.0160 |
| X-Mal-Mal Methylester | C7H6O6 | 186.0164 |
| X-Mal-Mal-Mal Methylester | C10H8O9 | 272.0168 |
| X-Glc-Mal-Mal | C12H14O11 | 334.0536 |
| X-Glc-Glc-Mal-Mal | C18H24O16 | 496.1064 |
| X-Glc-Glc-Glc-Mal-Mal | C24H34O21 | 658.1593 |
| X-Glc-Glc-Glc-Glc-Mal-Mal | C30H44O26 | 820.2121 |
| X-Glc-Mal-Mal-Mal | C15H16O14 | 420.0540 |
| X-Glc-Glc-Mal-Mal-Mal | C21H26O19 | 582.1068 |
| X-Glc-Glc-Glc-Mal-Mal-Mal | C27H36O24 | 744.1597 |
| X-Glc-Glc-Glc-Glc-Mal-Mal-Mal | C33H46O29 | 906.2125 |
| X-GlcA | C6H8O6 | 176.0321 |
| X-GlcA-GlcA | C12H16O12 | 352.0642 |
| X-GlcA-GlcA-GlcA | C18H24O18 | 528.0963 |
| X-GlcA-GlcA-GlcA-GlcA | C24H32O24 | 704.1284 |
| X-GlcA-Mal | C9H10O9 | 262.0325 |
| X-GlcA-GlcA-Mal | C15H18O15 | 438.0646 |
| X-GlcA-GlcA-GlcA-Mal | C21H26O21 | 614.0967 |
| X-GlcA-GlcA-GlcA-GlcA-Mal | C27H34O27 | 790.1287 |
| X-Glc-GlcA | C12H18O11 | 338.0849 |
| X-Glc-GlcA-GlcA | C18H26O17 | 514.1170 |
| X-Glc-GlcA-GlcA-GlcA | C24H34O23 | 690.1491 |
| X-Glc-GlcA-GlcA-GlcA-GlcA | C30H42O29 | 866.1812 |
| X-Glc-Glc-GlcA | C18H28O16 | 500.1377 |
| X-Glc-Glc-GlcA-GlcA | C24H36O22 | 676.1698 |
| X-Glc-Glc-GlcA-GlcA-GlcA | C30H44O28 | 852.2019 |
| X-Glc-Glc-GlcA-GlcA-GlcA-GlcA | C36H52O34 | 1028.2340 |
| X-Glc-GlcA-Mal | C15H20O14 | 424.0853 |
| X-Glc-GlcA-GlcA-Mal | C21H28O20 | 600.1174 |
| X-Glc-GlcA-GlcA-GlcA-Mal | C27H36O26 | 776.1495 |
| X-Glc-GlcA-GlcA-GlcA-GlcA-Mal | C33H44O32 | 952.1816 |
| X-Glc-GlcA-Mal-Mal | C18H22O17 | 510.0857 |
| X-Glc-GlcA-GlcA-Mal-Mal | C24H30O23 | 686.1178 |
| X-Glc-GlcA-GlcA-GlcA-Mal-Mal | C30H38O29 | 862.1499 |
| X-Glc-GlcA-GlcA-GlcA-GlcA-Mal-Mal | C36H46O35 | 1038.1820 |
| X-Glc-Glc-GlcA-Mal | C21H30O19 | 586.1381 |
| X-Glc-Glc-GlcA-GlcA-Mal | C27H38O25 | 762.1702 |
| X-Glc-Glc-GlcA-GlcA-GlcA-Mal | C33H46O31 | 938.2023 |
| X-Glc-Glc-GlcA-GlcA-GlcA-GlcA-Mal | C39H54O37 | 1114.2344 |
| X-Glc-Glc-GlcA-Mal-Mal | C24H32O22 | 672.1385 |
| X-Glc-Glc-GlcA-GlcA-Mal-Mal | C30H40O28 | 848.1706 |
| X-Glc-Glc-GlcA-GlcA-GlcA-Mal-Mal | C36H48O34 | 1024.2027 |
| X-Glc-Glc-GlcA-GlcA-GlcA-GlcA-Mal-Mal | C42H56O40 | 1200.2348 |
| X-GSH | C10H15N3O6S | 305.0682 |
| X-GSH+OH | C10H16N3O7S | 322.0709 |
| X-taurine | C2H5NO2S | 107.0041 |
| X-Glc-taurine | C8H15NO7S | 269.0569 |
| X-Mal-taurine | C5H7NO5S | 193.0045 |
| X-sulfate | O3S | 79.9568 |
| X-ethanolamine | C2H5N | 43.0422 |
| X-glycerol | C3H6O2 | 74.0368 |
| X-Ala | C3H5NO | 71.0371 |
| X-Arg | C6H12N4O | 156.1011 |
| X-Asn | C4H6N2O2 | 114.0429 |
| X-Asp | C4H5NO3 | 115.0269 |
| X-Cys | C3H5NOS | 103.0092 |
| X-Gln | C5H8N2O2 | 128.0586 |
| X-Glu | C5H7NO3 | 129.0426 |
| X-Gly | C2H3NO | 57.0215 |
| X-His | C6H7N3O | 137.0589 |
| X-Ile | C6H11NO | 113.0841 |
| X-Leu | C6H11NO | 113.0841 |
| X-Lys | C6H12N2O | 128.0950 |
| X-Met | C5H9NOS | 131.0405 |
| X-Phe | C9H9NO | 147.0684 |
| X-Pro | C5H7NO | 97.0528 |
| X-Pyl | C12H19N3O2 | 237.1477 |
| X-Ser | C3H5NO2 | 87.0320 |
| X-Thr | C4H7NO2 | 101.0477 |
| X-Trp | C11H10N2O | 186.0793 |
| X-Tyr | C9H9NO2 | 163.0633 |
| X-Val | C5H9NO | 99.0684 |

Table S3: Metabolites of AMT detected in different plant parts of cress and pea exposed to a 1mg L^-1^ solution over a period of 16 days. Values refer to average peak areas from measurements in triplicate.
